# The contribution of the socio-demographic characteristics on suicidal ideation among Israeli soldiers

**DOI:** 10.1186/s40696-016-0014-7

**Published:** 2016-03-01

**Authors:** Leah Shelef, Evyatar Ayzen, Nirit Yavnai, Eyal Fruchter, Orly Sarid

**Affiliations:** 1 Psychology Branch, Israel Air Force, Ramat-Gan, Israel; 2 Mental Health Department, Israel Defense Forces Medical Corps, Ramat-Gan, Israel; 3 IDF Medical Corps, Israel Defense Forces, Ramat-Gan, Israel; 4 Department of Social Work, Faculty of Humanities & Social Sciences, Ben-Gurion University of the Negev, Beer-Sheva, Israel

**Keywords:** Suicidal ideation, Suicide attempters, Severity adjustment difficulties, Soldiers

## Abstract

**Background:**

Suicidal ideation is a risk factor for suicide attempt. The aim of the present study is to compare suicidal ideation of different groups with different distress.

**Methods:**

100 soldiers, aged 18–21, divided into four research cohorts: soldiers who had carried out a suicide attempt (n = 40); soldiers with a psychiatric diagnosis (n = 20); soldiers having high severity adjustment difficulties (n = 20); and a control group of soldiers, having neither a history of mental health diagnosis, nor adjustment difficulties (n = 20). All completed the suicide ideation scale.

**Results:**

Half of the attempters had a psychiatric diagnosis (depression or anxiety) on the day of their enlistment and 37.5 % of them had a specified personality disturbance. The attempters were characterized by previously-attempted suicide (p < .01). The lowest mean values (*M* = 1.95, *SD* = .67) were among the attempter (F = 3.173, df = 3, p = .02) in motivation for military service. The variable expressing low motivation for military service was the sole predictor of suicide ideation (p = .032).

**Conclusions:**

Early diagnosis facilitated better monitoring by military mental health officers.

## Background

Suicide is a leading cause of death worldwide among adolescents and young adults [1]. Suicide attempts increase the scope of the phenomenon, and it most significant risk factors for death by suicide in adults [2–4]. A prevalent measure to estimate the risk for suicide attempts and completing suicide is assessing suicidal thoughts [5–7]. In the general population, the ratio between suicidal thoughts and completed suicides is 3:1 [8]. Some report that approximately 10–15 % of the general population experience suicide ideation for a week to a month during their lives [9].

The ratio of ideation to actual suicide attempts is much higher in adolescents than in adults, and 16.5 % of adolescents report actual suicide plans [10, 11]. The latency time and quantitative aspects of the relationship between suicide ideation and the actual execution of suicidal behavior or complete suicide has been the subject of considerable research [12–16].

There is a paucity of empirical study offering a multifactorial view on suicide ideation among suicide attempters/non-attempters and clinical/non-clinical subjects [17]. For example, a study with 196 participants found that negative life events had a significant effect on non-attempters but not on attempters; hopelessness moderately contributed to suicidal ideations among attempters but not among non-attempters, and loneliness contributed significantly to depression in non-attempters but was less distressing in attempters [17].

Several studies have identified numerous factors related to suicide ideation in adults and adolescents, including socio-demographic background, personality life stressors, distress symptoms, social support, alcohol use, and chronic conditions [18–21]. Findings from recent studies among soldiers [22] and veterans in the US army revealed that depression was positively associated with suicidal ideation [23].

Military service itself presents a stressful situation for many soldiers that could lead to suicidal thought [24]. One study in IDF examined the process leading to suicidal ideation, found that suicide attempters had higher levels of subjective stress as well as depression and hopelessness, compared to psychologically-treated and control groups [25].

There is still a substantial knowledge gap regarding the process of transition from suicidal thoughts to actions [15]. Despite the high frequency of suicide ideation in comparison to other suicidal behaviors, suicidal thoughts are perceived as reflective of a lower degree on the continuum of intensity of suicidal behaviors [26].

Further knowledge in this area could help identify factors contributing to suicidal thoughts and improve methods of suicide prevention among soldiers at risk. Thus, the purpose of this study was to deepen the knowledge about the contribution of various factors to suicidal ideation, by examining four different cohorts of soldiers in active IDF duty: soldiers having carried out a suicide attempt (SA), those having a psychiatric diagnosis (PD); those having adjustment difficulties (AD), and a control group with none of the above characteristics.

The originality of the current study is in its selection of comparison soldier cohorts: those having attempted suicide, those who had not made a suicide attempt but did have a psychiatric diagnosis (axis I, II; DSM-5) [27], and those who neither attempted suicide nor had a psychiatric or psychological diagnosis.

## Methods

### Participants

The sample included 100 soldiers on active duty (36 % females; 64 % males), aged 18–21 (*M* = 19, *SD* ± 1.0), mean length of service at the completion of the questionnaire was 12.7 months (*SD* = 8.36). The study population is presented in Tables 1, 2.

**Table 1:** Study population (*N* = 100)

Variables	SA^a^		PD^b^		AD^c^		C^d^		χ^2^
	Count	%	Count	%	Count	%	Count	%	
Demographic variables									
Gender									
Male	23	57.5	12	60.0	16	80.0	13	65.0	3.10 *df* = 1
Female	17	42.5	8	40.0	4	20.0	7	35.0	
Country of birth									
Israel	32	80.0	17	85.0	10	90.9	13	68.4	5.63 *df* = 2
Former soviet union	7	17.5	0	0	0	0	4	31.6	
Other	1	2.5	3	15.0	1	9.1	0	0	
Country of origin									
Israel	35	87.5	19	95.0	19	95.0	16	80.0	.74 *df* = 3
Former soviet union	4	10.0	1	5.0	0	0	4		
Ethiopia	0	0	0	0	1	5.0	0	0	
Other	1	2.5	0	0	0	0	0	0	
Socio demographic status									
High	13	33.4	5	25.0	2	10.5	8	40.0	.49 *df* = 2
Average	11	28.2	12	60.0	9	47.4	7	35.0	
Low	15	38.5	3	15.0	8	42.1	5	25.0	
Soldier’s status									
Single	3	7.5	0	0	1	5.0	4	20.0	2.87 *df* = 4*
Bereavement	0	0	4	20.0	2	10.0	0	0	
Severe AD^2^	1	2.5	0	0	0	0	0	0	
Special	5	12.5	0	0	1	5.0	0	0	
Normal	31	77.5	16	80.0	16	82.0	16	80.0	
Combat unit									
Yes	13	32.5	0	0	0	0	5	25.0	5.14 *df* = 1**
No	27	67.5	20	100	20	100	15	75.0	
Psychological variables									
Psychiatric diagnosis (enlistment day)									
Axis I	20	50.0	18	90.0	4	20.0	0	0	79.98 *df* = 1**
Axis II	15	37.5	0	0	0	0	0	0	
No diagnosis	5	12.5	2	10	16	80.0	20	100	
Medication									
Yes	7	17.5	9	45.0	4	20.0	0	0	12.96 *df* = 1**
No	33	82.5	11	55.0	16	80.0	20	100	
Previous psychological therapy									
Yes	13	32.5	10	50.0	7	36.8	8	40.0	1.76
No	27	67.5	10	50.0	12	63.2	12	60.0	
Previous suicide attempt									
Yes	14	35.0	2	10.0	2	10.0	0	0	13.62 *df* = 3**
No	26	65.0	18	90.0	18	90.0	19	100	
ADHD									
Yes	8	20.0	3	15.0	11	55.0	9	49.4	12.35 df = 3**
No	32	80.0	17	85.0	9	45.0	10	52.6	
Drug use									
Yes	2	5.0	1	5.0	1	5.0	2	10.0	.70 ns
No	38	95.0	19	95.0	19	95.0	18	90.0	
Alcohol use									
Yes	9	22.5	0	0	1	5.0	0	0	11.94 *df* = 3**
No	31	77.5	20	100	19	95.0	20	10	
Criminal record									
Yes	3	7.5	0	0	1	5.0	0	0	2.99 ns
No	37	92.5	20	100	19	95.0	20	100	
Social support Variable									
Romantic relationship									
Yes	5	12.8	6	30.0	6	30.0	6	30.0	3.91 *df* = 3 ns
No	34	87.2	14	70.0	14	70.0	14	70.0	

Fisher’s exact test *p* < .001***

^a^ Attempted suicide

^b^ Psychiatric diagnosis

^c^ Adjustment difficulties

^d^ Control

**Table 2:** Continuous variables One-way ANOVA tests (*SD, M, F*)

Variables	AS^a^		PD^b^		AD^c^		C^d^		*F* (*df* = 3, 96)
	*M*	*SD*	*M*	*SD*	*M*	*SD*	*M*	*SD*	
Demographic variables									
Education	11.83	.87	11.95	.22	11.70	.92	11.95	.22	.585 ns
Duration of military service	12.35	8.23	12.10	9.11	10.65	5.96	13.40	10.11	0.364 ns
Psychological variables									
Intellectual rating score	47.25	20.99	51.0	14.83	38.5	17.25	43.5	13.48	1.87 ns
Motivation for service	1.95	0.67	2.35	0.67	2.15	.58	2.45	.68	3.17*
Family variable									
Perceive family support									
Mother	2.17	.78	2.60	.59	2.15	.74	2.50	.76	2.23 ns
Father	1.94	.85	2.11	.83	2.0	.88	2.26	.93	.60 ns
Social support variable									
Perceive social support (in the army)	2.33	.77	2.10	.85	1.85	.74	2.15	.81	1.68 ns
Perceive social support (at home)	1.82	.71	1.70	.57	1.40	.59	1.90	.71	2.35 ns
Independent variables									
Suicidal ideation	15.5	11.38	5.55	8.03	6.30	8.82	3.70	8.50	9.32**

* *p* < .05; ** *p* < .01

^a^ Attempted suicide

^b^ Psychiatric diagnosis

^c^ Adjustment difficulties

^d^ Control

The study population was divided into four defined cohorts:

- **Suicide Attempt (SA).** The main research group comprised 40 soldiers who had attempted suicide during the three months prior to completing the questionnaire, and were hospitalized for at least 24 h in wake of this attempt.
- **Psychiatric Diagnosis (PD).** The PD group comprised 20 soldiers bearing a recognized axis I psychiatric diagnosis, but with no known suicide attempts.
- **Adjustment Difficulties (AD).** The AD group comprised 20 soldiers diagnosed as having minor adjustment difficulties, as documented in their medical records by a mental health professional as a guide in assigning them to a suitable military placement [28]. None of the AD soldiers had executed a known suicide attempt prior to their inclusion in the study. Adjustment difficulties are defined as a combination of personality traits limiting functionality and adaptability for military service. This designation is not to be confused with adjustment disorders as reflected in the DSM.
- **Control Group (CG).** The CG group comprised 20 soldiers bearing no known psychiatric pathology, adjustment difficulties, or any previous suicide attempt. This group included soldiers who met with a psychologist in the past, and were evaluated, but were not found to have a psychiatric diagnosis or any marked adjustment or psychiatric difficulties. The rationale for identifying these soldiers as controls was their having been previously assessed by a mental health clinician, and relevant data was available.

Military service in Israel is compulsory for most boys and girls, aged 18–21 [29]. During the enlistment process the conscripts are screened for physical and psychiatric disorders [30]. They are queried regarding psychiatric history, mental crises, as well as suicidal thoughts. Those suspected of mental illness or suicide risk are referred for a psychiatric examination [31]. Those having a less severe emotional disturbance are able to complete their army enlistment, but will have this status documented in their army medical records, in order to help mental health officials monitor and treat them as needed [31].

### Materials and procedure

Data was gleaned from IDF’s computerized database, which includes soldiers’ characteristics (e.g., recruitment date, military occupation, intellectual rating score) and mental health unit data. All diagnostic evaluations and treatment meetings conducted by a mental health officer are documented in the computerized patient record (CPR). The CPR holds all the soldier’s current and past medical records. When a soldier engages in self-injury or executes a suicide attempt during his military service, the health officer files a report assessing the lethality and severity of the suicide behavior.

Data collection took place from June 2012 to February 2014 in two stages: In the first stage, data were collected regarding soldiers who had carried out a suicide attempt. Within this period, 81 soldiers were hospitalized after attempting suicide, of which 32 were discharged as being unfit for further military service. Inclusion criteria for the SA group were: being hospitalized for at least 24 h for treatment or for observation after attempting suicide [32] and being evaluated as high risk by a mental health officer.

The remaining 49 soldiers, while resuming their military service, were recognized by mental health officers as being high risk for suicide, sharing these soldiers’ statuses with one of the study researchers. A research assistant contacted each of the 49 soldiers and invited them to participate in the study, following the protocol approved by the ethics committee, with 40 of them completing the study questionnaires.

In the second stage, data were collected from the three comparison groups. A quota sampling was conducted according to the following procedure: Each mental health officer who had treated a soldier having executed a suicide attempt and participating in the first stage, identified three additional soldiers that met the following criteria: psychiatric diagnosis (PD group; *n* = 20), severe adjustment difficulties (AD group; *n* = 20) and control group (CG; *n* = 20).

The study was approved by the IDF Human Research Review Board. In addition the study recruitment team did not have any relationship with the participants’ units.

### Measures

#### Suicide ideations

This 21-item self-report questionnaire examines suicidal ideation [33]. It was designed to examine two factors: the degree of seriousness of the desire to die and of the suicide intention. Each question category describes a different level of severity of suicide ideation on a 3-point Likert-type scale, ranging from a low degree of suicide ideation (0) to a high degree of suicide ideation (2). Examples: *I have a moderate to strong desire to live; I have little desire to live; I have no desire to live.* The final score was procured by summing the item categories for severity of suicide ideation. In this manner, a higher score reflects a higher intensity of suicide ideation. Cronbach’s alpha coefficient in the current study was α = .90.

### Demographic variables

*Socio*-*demographic data* were retrieved using the IDF Human Resource database that includes the following variables: gender, country of birth, father’s country of origin, and years of education.

*Socioeconomic status* was determined by a residence location score: low (1–3), average (4–6), and high (7–10). The scale was created by dividing the country into residential clusters, based on a classification by Israel’s Central Bureau of Statistics (CBS, 2006) and numbering them from 1–10, with higher numbers corresponding to a higher socioeconomic status.

#### Soldiers’ status

Includes several variables, describing the level of the soldiers’ relationship and support derived from their family, an important resource that can moderate military-related stress. These criteria were (1) *Lone soldiers*: soldiers whose family lives outside the country and unavailable for direct support during the soldier’s military service. (2) *Bereaved soldier*: Soldiers belonging to a family where one of its members (father or brother) was killed while on reserve or active duty. (3) *Severe adjustment difficulties*: soldiers carrying a diagnosed emotional or behavioral disturbance. (4) A special category of soldier, whose family lives in Israel but, for various reasons, there is no contact between them. (5) Regular soldiers having no known family issues.

#### Combat duty

At enlistment, each conscript is assigned a medical profile score, indicating physical and mental fitness as well as limitations (e.g., visual and auditory levels). The profile classification of the soldier determines their assignment to specific units, including combat and non-combat ones [28].

*Duration of military service*—measured by the number of months in the military at the time of the data collection.

### Psychological variables

During the enlistment process the candidates are interviewed and evaluated. If emotional or behavioral disturbances are suspected, the subject is referred to a clinical social worker or clinical psychologist, who conducts a semi-structured interview, followed by further evaluation, as needed [34]. All soldiers participating in the study had complete Medical and Mental Health Records.

*Psychiatric diagnosis*—the medical profile is comprised of a psychiatric evaluation, based on the ICD-10 [35], and an assessment of its impact on the soldier’s potential functioning [34]. In our study we divided the diagnosis by Axis I and Axis II, as common in the DSM-5 [27].

*Adjustment difficulties*—a measure, determined by an IDF mental health officer, and used for assessing the soldier’s potential adjustment difficulties in military service (ranging from 00–50); in our study we adopted the severe adjustment score (43 or 50).

*Intelligence*—intellectual ability was measured by the intellectual rating score (IRS) [34]. The total score is regarded as equivalent to a normally-distributed IQ score [28, 29]. Participants were classified into three categories by IRS score: low (10–30), average (40–60) and high (70–90).

*Motivation during military service*—The motivation of soldiers is assessed during the mental health officer’s clinical examination by means of a single question: What is the soldier’s declared level of motivation to continue in military service? Answers are given on a 5-point scale: (a) *does not want to serve at all*. (b) *will serve in the army only because it compulsory*. (c) *will serve only if the conditions suit him/her*. (d) *wants to serve, but not in combat*. (e) *wants to serve in combat.*

The remaining psychological variables were assessed by questions, limited to a yes/no response: previous psychological therapy, prescribed medication, previous suicide attempt, deficit hyperactivity disorders (ADHD), drug and alcohol use, and having a criminal record.

### Family variables

*Family support* was assessed by two questions, regarding the soldier’s relationship with his parents. The therapists needed to distinguish between a supportive relationship with mother/father, good relations with mother/father, and relationship limited to concretes needs.

*Quality of the relationships* with parents was rated on a 3-point scale: high, moderate, low.

### Social variables

These variables included: *perceived romantic relationship* and *peer group social support at home and in the army*. (*Do you have any romantic relationship with girl/boy friend*, yes/no)?

Quality of the relationships they have with friend at home or in the army, were evaluated on a 3-point rating scale: high, moderate, low.

### Statistical analysis

SPSS, version 20.0 for Windows, was used for all analyses.

To examine the distribution of soldiers according to their demographic, psychiatric, family, and social characteristics, tests were conducted for categorical variables. Comparison of demographic, psychological, family and social support continuous variables between the study groups was conducted using one-way ANOVA.

Tukey HSD test was employed to examine suicidal thoughts between the four groups. Finally, a linear regression model was constructed for predicting suicide ideation as the dependent variable. The level of statistical significance was set to *p* = .05.

### Ethics approval

The Institutional Review Board of IDF Medical Corps approved the study and waived the requirement for informed consent on the basis of preserving participants’ anonymity.

## Results

The study’s first aim was to examine the distribution of soldiers, by demographics, psychiatric diagnosis, family status, and social characteristics. Percentages and χ^2^ for categorical variables are presented in Table 1.

The four groups of soldiers differed significantly in only a few variables. The control group included 7.5 % *(n* = 3) lone soldiers, with the PD group including 20 % (*n* = 4) who were members of a bereaved family (*χ* ^2^ = 2.87, *df* = 4, *p* < .05). As for the combat variable, as expected, no soldiers in the PD and AD groups had served in combat units, while 32.5 % of the SA group and 25 % of the control group had served in combat units (χ^2^ = 5.14, *df* = 1*, p* < .01).

Higher levels of medication use was reported in the PD group (45 %, χ^2^ = 12.96, *df* = 1, *p* < .01), as shown in Table 1. More psychiatric diagnoses on both axes I (depression or anxiety) and II (personality) were found on enlistment day for the SA group. Half of the SA soldiers (*n* = 20) had a diagnosis on Axis I and 37.5 % (*n* = 15) had a diagnosis on axis II (χ^2^ = 79.98, *df* = 1, *p* < .01). Ninety per cent of the PD group had a specific axis I diagnosis (*n* = 18), and the remaining had a unspecified psychiatric diagnosis.

It is noteworthy that 35 % of the SA group reported a previously attempted suicide, mostly undocumented in the mental health history records (*n* = 14, χ^2^ = 13.62, *df* = 3, *p* < .01).

Significant differences were found for attention deficit hyperactive disorder (ADHD), 55 % of the AD group and 47.0 % of the control group reported having ADHD, whereas only 20 % of the SA group and 15 % of PD group reported ADHD (χ^2^ = 12.35, *df* = 3, *p* < .01).

As for self-reported alcohol use, 22.5 % of the SA group reported use of alcohol, a rate significantly higher than that reported in the other three groups (χ^2^ = 11.94, *df* = 3, *p* < .01).

One-way ANOVA tests were conducted to compare continuous variables (see Table 2). Statistically significant differences were found at the level of motivation to military service: highest mean values of motivation levels characterized the control group (*M* = 2.45, *SD* = .68) and the lowest mean values (*M* = 1.95*, SD* = .67) characterized the SA group (*F* = 3.173, *df* = 3, *p* = .02).

As for suicide ideation, one-way ANOVA revealed a statistically significant difference between the SA groups compared to the other three groups: SA (*M* = 15.5, *SD* = 11.38), PD (*M* = 5.55, *SD* = 8.03), AD (*M* = 6.30, *SD* = 8.82), with the control group reporting the lowest mean values (*M* = 3.70, *SD* = 8.50, *F* = 9.32, *df* = 3, *p* ≤ .01).

Finally, in order to answer the remaining study aim, regarding identifying the variables that contribute to suicidal ideation, two linear regression tests were conducted, with suicidal ideation score as the dependent variable. The first linear regression was conducted with all four research groups as independent variable. Variables found significant in the univariate analysis (see Table 1) were entered as subsequent independent variables: soldier’s status, combat duty, psychiatric diagnosis, medication, previous psychological therapy, ADHD, alcohol, and motivation for service. Motivation for service was inversely related to suicidal ideations, (β = −9.611, *p* = .02) and explained 25.6 % of the variance (see Table 3).

**Table 3:** Linear regression model among the research population, R^2^ = .256 (n = 100)

Variables	Unstandardized coefficients			Confidence interval for B		
	B	SD	Beta	Sig	LB	UB
Constant	20.607	6.83		.005	6.70	34.51
lone soldier	3.98	7.08	.09	.578	−10.45	18.41
Bereavement	–	–	–	–	–	–
Combat unit	5.0	4.07	.21	.229	−3.30	13.30
Psychiatric diagnosis	−.18	5.93	−.010	.976	−12.25	11.90
Medication	−6.61	5.87	−.19	.269	−18.57	5.35
Previous suicide attempt	−.27	4.809	−.001	.946	−8.46	7.91
ADHD	7.22	4.52	.25	.120	−1.99	16.42
Alcohol	−.1.45	4.77	−.05	.762	−11.16	8.25
Motivation for service	−9.61	4.08	−.37	.025	−17.92	−1.30

A second linear regression test was conducted only for the SA group. Four statistically significant variables were entered into this model (Table 1): previous suicide attempt, alcohol use, motivation, and psychiatric diagnosis (Table 4). As in the first examination, motivation for military service was the sole predictor of suicide ideation among the suicidal group (β = −.356, SE = 4.139, *p* = .03) and added 13.1 % to the explained variance (Table 4).

**Table 4:** Linear regression model among suicidal group, R^2^ = .131 (n = 40)

Variables	Unstandardized coefficients			Confidence interval for B		
	B	*SD*	Beta	Sig	LB	UB
Constant	22.55	6.819		.002	8.715	36.400
Psychiatric diagnosis	−.342	5.859	−.010	.954	−12.236	11.552
Motivation for service	−9.242	4.139	−.356	.032	−17.644	−.840
Previous suicide attempt	.221	3.809	.009	.954	−7.512	7.955
Alcohol	.758	4.540	.028	.868	−8.459	9.975

## Discussion

The purpose of this study was to deepen the knowledge about the contribution of various factors to suicidal ideation, by looking at four different cohorts of soldiers in active IDF duty: soldiers who carried out a suicide attempt (SA), those having a psychiatric diagnosis (PD); those having adjustment difficulties (AD), and a control group, having none of the above characteristics.

The PD group (soldiers with a psychiatric diagnosis-especially depression and anxiety) was fully non-combatant (100 %), used more psychiatric medication during their military service (45 %), and experienced greater bereavement in their family (20 %). A possible explanation for these findings is their mental health status assessed at induction, which led to their being assigned to non-combatant units [28]. About half of these soldiers used medication, compared to less than a fifth of those in the SA or AD groups. The high percentage of bereavement within this group is consistent with previous studies showing links between the loss of a family member and having a psychiatric diagnosis [36–38]. Despite the psychiatric diagnosis, participants in this group had lower levels of suicide ideation compared to soldiers in the AD and SA groups, perhaps due to the continuous support and treatment given by mental health officers and medication.

Regarding the AD group, more than half had been diagnosed with ADHD, compared with about 20 % of the SA, 20 % of the control group, and 15 % of the PD group. AD soldiers are likely to have educational, social, and psychological difficulties [39]. Their psychosocial profile is characterized by emotional, behavioral, and cognitive problems [40]. The IDF’s emotional disturbance diagnosis, determining the AD group classification, includes an intense need for attention, difficulty delaying gratification, impulsiveness, a low frustration threshold, difficulty establishing trust and accepting responsibility, and low self-esteem [28, 39]. Some of the above features are consistent with the ADHD diagnosis. Soldiers with ADHD and adjustment difficulties are viewed as having undeveloped problem-solving skills, an impaired ability to plan ahead, difficulty functioning under stress and accepting authority, and have difficulty taking initiative and responsibility [28]. After the SA group, the AD group had the lowest level of motivation for army service and least suicide ideation, compared the PD and control groups. The fact that soldiers with AD received rigorous treatment and follow up may explain their low level of suicide ideation.

The soldiers in the control group have specific characteristics: a relatively high 20 % are lone soldiers, with about half of these reporting having an ADHD diagnosis. This is an interesting finding, considering that the percentage of ADHD in the general population approximates 15–20 %. It may be that more ADHD-diagnosed soldiers volunteer to a combat unit, given the units’ high action orientation, since serving in a non-combat role would likely place them in an office environment, where their learning difficulties would prove a liability. The high percentage of lone soldiers in combat units is intriguing since this placement is discretionary for them in the IDF. The challenge facing a combat soldier, having to cope with combat-related stress along with having less available social support from their immediate families, is cited in the literature as imposing a negative influence on the soldier’s individual performance and mental strength [41]. The interpersonal theory [15], emphasizing the importance of a sense of belonging to one’s mental well-being and effective coping with stressors and life events [38, 42], may explain this finding. It may be that the social support provided by peer soldiers in the combat unit, the unit’s high level of group cohesion, the sense of belonging, and pride in being a member of a combat unit provide a possible explanation for the ability of lone soldiers to cope with the challenges associated with their difficult service. Another possible explanation can be their high level of motivation to serve. Since a lone soldier can elect not to be in a combat unit, those choosing to serve in a combat unit would likely have mostly high motivation to serve. High motivation and belief in one’s purpose are considered in the literature as personal resources that facilitate coping with military-related challenges [43, 44].

Our findings corroborate those of other studies showing that individuals having attempted suicide suffer from mental disorders and/or personality disorders [45, 46]. Previous suicide attempt is a known predictor for suicide attempts and completion [47–49]. These findings are consistent with those of a previous study conducted in the IDF that showed 37.9 % of the soldiers who attempted suicide during their service had a previous pre-army suicide attempt, and about a fifth reported self-harm behaviors [24].

Soldiers who attempted suicide reported significantly higher alcohol use. Alcohol use has been positively associated with suicide acts. These links were found among adolescents [48], US soldiers [50, 51], and among IDF soldiers [24]. Previous studies also found an association between alcohol use and mental illness, such as anxiety disorders, mood disorders, and depression [46, 52]. For example, findings from an earlier IDF study, conducted among soldiers who attempted suicide during their military service, showed that about half had been diagnosed with a personality disorder, while 8.6 % were diagnosed with mood disorders [24]. Alcohol use was two times more prevalent among the suicide attempters [24].

Several limitations of this study need to be noted. The first limitation concerns the relatively small sample and the method of quota sampling for each of the study groups. Future research would benefit from recruiting a large cohort sample of soldiers. The second limitation refers to the examined variables. We recommend that future studies examine a wider range of self-report scales, such as functioning, well-being, distress levels, and suicidal ideation. A third limitation may be that soldiers characterized by low levels of motivation to serve, submitted answers reflecting a denial or an embellishment of their actual condition. Future research could adopt a mixed method with the military sample, including self-report questionnaires as well as in-depth interviews. A final limitation relates to the generalizability of the military study population, which necessarily excludes those not serving. For instance, when a soldier’s situation is deemed very acute following a suicide attempt, and upon considering all relevant variables, the soldier is almost immediately discharged from his military service. In this situation, these soldiers were inaccessible subsequent to their discharge.

## Conclusions

Low levels of motivation for army service impose a major risk factor for suicide ideation, found significantly lowest in the SA group. We hypothesized that SA soldiers would have greater suicidal ideation compared to the other three soldier groups in our study. One comparison group was characterized by adjustment difficulties (AD) and the other with psychiatric disorders (PD), which also constitute a significant risk factor for suicidal ideation [45, 46, 53]. These two groups are identified and classified prior to induction or during their service.

Early diagnosis may have enabled better monitoring of the soldiers, such as provision of therapeutic sessions and professional surveillance by military mental health officers. Support for this can be found in a previous study demonstrating that soldiers being treated by professionals throughout their military service had low rates of suicide attempts [28].
